# Peptide Sharing Between Viruses and DLX Proteins: A Potential Cross-Reactivity Pathway to Neuropsychiatric Disorders

**DOI:** 10.3389/fnins.2018.00150

**Published:** 2018-03-21

**Authors:** Guglielmo Lucchese, Benjamin Stahl

**Affiliations:** ^1^Brain Language Laboratory, Freie Universität Berlin, Berlin, Germany; ^2^Department of Neurology, Universitätsmedizin Greifswald, Greifswald, Germany; ^3^Department of Neurology, Charité Universitätsmedizin Berlin, Berlin, Germany; ^4^Department of Neurophysics, Max Planck Institute for Human Cognitive and Brain Sciences, Leipzig, Germany; ^5^Psychologische Hochschule Berlin, Berlin, Germany

**Keywords:** viral infections, neuropsychiatric diseases, language disorders, fetal and adult neurogenesis, DLX proteins, peptide sharing, cross-reactivity, autoimmunity

## Abstract

The present study seeks to determine potential associations between viral infections and neuropsychiatric diseases. To address this issue, we investigated the peptide commonalities between viruses that have been related to psychiatric and neurological disorders—such as rubella, human immunodeficiency virus, and herpesviruses—and human distal-less homeobox (DLX) proteins expressed in developing brain—namely, DLX1, DLX2, DLX5, and DLX6. Peptide matching analyses revealed a high degree of pentapeptide sharing. From an immunological perspective, this overlap is relevant because pentapeptides are endowed with immunogenicity and antigenicity—that is, they are immune determinants. Moreover, infection-induced immune cross-reactions might have functional, spatial, and temporal implications related to the functions and expression patterns of DLX1 and DLX5 in the fetal and adult human brain. In sum, our data support the hypothesis that viral infections may be linked to neuropsychiatric diseases through autoimmune cross-reactions caused by molecular mimicry between viral proteins and brain-specific DLX self-antigens.

## Introduction

Infections, neuropsychiatric diseases, and language disorders are often concomitant pathological events that can have early etiological roots in fetal life and then become apparent in any stage across the life span of the individual, from the postnatal period to the late age (Yolken and Torrey, 1995; Coplan et al., 1998; Arias et al., 2012; Brown, 2012, 2015; Khandaker et al., 2013). However, it is unclear how these pathological events are mechanistically interlinked and temporally related, most likely because the wide spectrum of infectious agents and the varying extent of the numerous neuropsychiatric symptoms make it difficult to dissect the molecular correlations between infections and brain damage (Ludlow et al., 2016).

During the past few decades, scientific-clinical research examined the assumption that infections may relate to neuropsychiatric disturbances through autoimmune mechanisms (Knuesel et al., 2014; Severance et al., 2014; Blomström et al., 2015; Estes and McAllister, 2016; de Haan et al., 2017). More recently, it was suggested that anti-pathogen immune responses cross-reacting with the human NMDA receptor 2A subunit—alterations of which are involved in language dysfunctions (Carvill et al., 2013; Turner et al., 2015)—might represent a pathologic background for infections and many neurodegenerative disorders, ranging from schizophrenia to frontotemporal dementia (Lucchese, 2016).

The current study is extended to four members of the DLX transcription factor (TF) family—namely DLX1, DLX2, DLX5, and DLX6—that have been thoroughly investigated in numerous studies on neurodevelopment. Indeed, the four TFs are expressed during early fetal neurodevelopment (Merlo et al., 2000; Panganiban and Rubenstein, 2002) and are associated with the specification of γ-aminobutyric acid (GABA)ergic interneurons in the vertebrate forebrain subventricular zone (SVZ) as well as with granule neurons in the subgranular zone (SGZ) (Simeone et al., 1994; Anderson et al., 1997a,b). The issue appears to be important especially when considering that cognitive and emotional tasks occur in the neurogenic areas (Aimone et al., 2011; Ming and Song, 2011; Miller and Hen, 2015), and that altered adult neurogenesis and hyppocampal lesions have been repeatedly related to neuropsychiatric conditions (Parent and Murphy, 2008; Gonçalves et al., 2016; Inta et al., 2016; Kang et al., 2016; Yun et al., 2016; Kohman and Rhodes, 2017) and language disturbances (Sass et al., 1992; MacKay et al., 1998; Covington and Duff, 2016; Piai et al., 2016).

In this context, we hypothesized that an infection-induced maternal immune response may cross-react with DLX proteins, thus possibly causing a first subclinical immune-mediated damage of the developing nervous system. Later, successive encounters in adulthood with pathogens able to induce cross-reactions with DLX proteins might further damage regions of the adult brain (the subventricular zone and dentate gyrus), where DLX proteins may be expressed (Lim and Alvarez-Buylla, 2016) thus triggering the onset of neuropsychiatric clinical manifestations.

Focusing on infections as a trigger of DLX alterations and seeking for a possible mechanism, we proceeded along three steps. Firstly, we investigated whether or not infectious pathogens have the molecular basis to react with human DLX proteins—that is, we searched for shared peptides that might lead to cross-reactions. Secondly, we analyzed the immunological potential of the viral vs. DLX peptide overlap. Thirdly, we collected data on the expression level of DLX proteins in the fetal and adult human brain.

## Methods

The primary amino acid (aa) sequences of human DXL1 (Uniprot: P56177, 256 aa), DLX2 (Uniprot: Q07687, 328 aa), DLX5 (Uniprot: P56178, 289 aa), and DLX6 (Uniprot: P56179; 175 aa) were dissected into pentapeptides offset by one aa residue: MTMTT, TMTTM, MTTMP, and so forth. Then, each of the resulting pentapeptides was analyzed for matches to a sample library of 25 viral proteomes using the Protein Information Resource (PIR) match program (https://research.bioinformatics.udel.edu/peptidematch/batchpeptidematch.jsp) (Chen et al., 2013), as previously described (Kanduc et al., 2008; Lucchese et al., 2014; Lucchese, 2016, 2017). Neuronal Regeneration-Related Protein (NREP, Uniprot: Q16612, 68 aa) was used as a control neural protein. Glutamate decarboxylases 1 (Uniprot: Q99259; GAD-67; 594 aa) and 2 (Uniprot: Q05329; GAD-65; 585 aa) were additionally investigated as DLX-related proteins.

The virus library consisted of 25 proteomes derived from the viruses listed as follows (with Abbreviation, NCBI Tax ID, number of proteins, and aa length in parentheses): Borna disease virus (BDV; 928296; 6 proteins; 3014 aa); Epstein-Barr virus (EBV; 82830; 56 proteins; 30727 aa); Hendra virus (HeV; 928303; 9 proteins; 6956 aa); Hepatitis B virus genotype C subtype ayr (HBV-C; 928302; 5 proteins; 1792 aa); Hepatitis C virus genotype 1a (HCVH; 11108; 11 proteins; 3011 aa); Human cytomegalovirus (HCMV; 295027; 168 proteins; 63460 aa); Human herpesvirus 1 (HHV1; 10299; 73 proteins; 38368 aa); Human herpesvirus 2 (HHV2; 10315; 72 proteins; 38122 aa); Human herpesvirus 6A (HHV6A; 10370; 101 proteins; 43629 aa); Human herpesvirus 6B (HHV6B; 36351; 98 proteins; 43692 aa); Human immunodeficiency virus type 1 group M subtype A (HIV-1; 11697; 9 proteins; 3585 aa); Human parvovirus B19 (HPV-B19; 648237; 5 proteins; 1701 aa); Influenza A virus, H5N5 (IVA, H5N5; 93838; 12 proteins; 4809 aa); Influenza A virus, H1N1 (IVA, H1N1; 211044; 12 proteins; 4788 aa); Influenza A virus, H7N7 (IVA, H7N7; 384493; 12 proteins; 4802 aa); Influenza B virus (FLUBV; 518987; 11 proteins; 4718 aa); Influenza C virus (FLUCV; 11553; 8 proteins; 4259 aa); Measles virus (MeV; 645098; 8 proteins; 5202 aa); Rotavirus A (RV-A; 450149; 12 proteins; 5897 aa); Rotavirus C (RV-C; 31567; 11 proteins; 5608 aa); Rotavirus X (RV ADRV-N; 335103; 11 proteins; 5679 aa); Rubella virus (RUBV; 11045; 5 proteins; 3179 aa); Vaccinia virus (VACV; 10254; 217 proteins; 56795 aa); Varicella-zoster virus (HHV-3; 10338; 69 proteins; 35782 aa); and Zika virus (ZIKV; 64320; 13 proteins; 3419 aa).

The peptide sharing was investigated for immunologic potential using the Immune Epitope Database (IEDB; www.iedb.org) resource (Vita et al., 2015). Epitopes that have been experimentally validated as immunopositive in the human host were considered.

Laser microdissection microarray data on DLXs and NREP transcript expression in fetal and adult human brain were obtained from the online database of the Allen Institute for Brain Science (http://www.brainspan.org/; http://human.brain-map.org/) (Lein et al., 2007; Miller et al., 2014). Data on DLX and NREP protein expression in adult human brain were retrieved from https://www.proteinatlas.org/humanproteome (Uhlén et al., 2015; Thul et al., 2017).

## Results and discussion

A sample library formed by 25 virus proteomes was analyzed for pentapeptide sharing with DLX proteins. Pentapeptides were used as probes, for five aa residues represent a minimal immune-biological determinant in humoral and cellular immune recognition (Kanduc, 2012, 2013 and further refs. therein). NREP, a protein involved in neuronal regeneration (Fujitani et al., 2004), was used as a neural control protein.

The analyzed viral set consisted of pathogens that have been reported in studies on language disorders and other cognitive dysfunctions, bipolar disorders, and schizophrenia (Yolken and Torrey, 1995; Coplan et al., 1998; Brown et al., 2004, 2006; Torrey et al., 2006; Baillargeon et al., 2008; Buka et al., 2008; Mortensen et al., 2010; Arias et al., 2012; Brown, 2012; Hornig et al., 2012; Khandaker et al., 2013; Blomström et al., 2015; Canuti et al., 2015; Lucchese, 2016; Ludlow et al., 2016; Soltani et al., 2016).

### Pentapeptide sharing between DLX proteins and potential viral pathogens

The quantitative and qualitative pentapeptide overlap between the four human TF DLX proteins and NREP vs. the set of 25 virus proteomes is shown in Table 1.

**Table 1:** Pentapeptide sharing between 25 viral proteomes and the human DLX and NREP proteins.

**Virus**	**Viral pentapeptides shared with**				
	**DLX1 (94; 37%)**^a^	**DLX2 (156; 48%)**^a^	**DLX5 (96; 34%)**^a^	**DLX6 (35; 21%)**^a^	**NREP (13; 20%)**^a^
BDV	–	–	–	–	–
EBV	ESLNS; PGADS; PPVPP; RKPRT; SASSF; SFSRP; SLNSP; SSASS; SSLQL; VVEGG	AALQR; AGGGG; ASGLN; DFGVP; GGGAG; GGGGP; GPGGN; GSGGS; GSSGS; HLQAT; NSSSS; PVSTA; QATAP; QQPPS; RKPRT; SAKSS; SGGGA; SGGSG; SHLQA; SSPSS; SSSSS; VRKPR	AALQR; KAYAD; PTSAS; RKPRT; SPQSP; SPSSS; SPTSA; VRKPR	MQRPQ; RKPRT; RSPAL; SAALS; SSLQL	AASLT
HeV	AELAA	AELAA; SSTYH	AELAA	AELAA	–
HBV-C	–	–	SPSSS	–	–
HCVH	GGNAG; PPVPP; SPPVP	VSTAT	–	GGNAG; PPVPP; SPPVP	NAASL
HCMV	ASLGL; EGSAL; GGEVR; GNAGS; GRALS; KAVFM; LAASL; LEGSA; MSPSP; NSSSG; NSVSS; PPGWN; PPVPP; PVPPG; QALNR; RLEDP; SHASS; SSASS	AASST; AGGGG; ASLGL; EQHPG; GGGAG; GGGGA; GGGGG; GGGGS; GSGGS; HGGGG; HHHGG; HHHHH; IAASS; LAASL; LPVST; NSSSS; PGASA; PGSGG; PPVSA; PSSAA; PVSAP; PVSTA; PYGTS; QQPPS; SAFLG; SASHL; SGGGA; SGGSG; SPPVS; SSGSS; SSPSS; SSSSL; SSSSS; VSAPA	AASSI; AKAYA; ASLGL; ASSYL; FQTSA; HPPTS; HSPSS; LAASL; PQGSS; RRVPS; SAASS; SPSSS; SYASS	AALSP; ASLGL; LAASL; LKQGS; SSAAA	GRLPV; LTPLG; SLTPL; SSELR; TNAAS
HHV1	EDPGA; GEVRF; GGAAL; GNAGS; PGSAS; PPVPP; SHASS; SSASS; VEGGE; YIPSY	AALQR; DSLVA; GAGPG; GGAGP; GGGGP; GGGGS; GGSGA; GSGAG; PAGGG; PGASA; PGSGG; PPVSA; PSSAA; PVSAP; SAASA; SGAGS; SSAAS; SSSPA; SSSSS; TSSSP; VSAPA; VSTAT; YQASG	AALQR; GAPHG; KRSKI; LASGT; PAVWE; PQSPA; RSKIK; SAASS; SSSDP	SPALP; SSAAA	AASLT; LTPLG; SLTPL
HHV2	SSASS; SASLA; VEGGE; GEVRF; GGAAL; AALEG; PPVPP; GNAGS	AALQR; AGGGG; AGSSG; ASASP; DSLVA; GAGPG; GGAGP; GGGAG; GGGGA; GGGGG; GGPGS; GGSGA; HHHHH; PGASA; PSSAA; SLVAD; SSSSS; TLPVS; VSTAT	AALQR; ALNPY; ATDSD; HAHPP; HHPHA; KRSKI; LPPPG; PAVWE; PPPGS; PQSPA; RSKIK; RVPSA; RVPSA; SPASS; SPTSA; YPAKA	PALPP; SSAAA; VSASA	AASLT
HHV6A	AELAA; ASLAQ; ASSFS; GSALA; PPVPP; SEKST; SGGNA; SKFKK; SSASS; VSGKA	AELAA; LNNVP; PCASP; PVSTA; RRFQK; SKFKK; SSSSS	AELAA; EVTEP; GSSRS; KEVTE; RRFQK; SRSLS; YASSY	AELAA; KLLKQ; SAALS; SKFKK; SPRSP	AASLT; EGRLP; LTPLG
HHV6B	ASLAQ; ASSFS; GSALA; QQPQL; SGGNA; SKFKK; SSASS; VRFNG; VSGKA	KTQYL; LNNVP; PCASP; PVSTA; RRFQK; SGEIP; SKFKK; SSAAS; SSSSS	KEVTE; KTQYL; RRFQK; SRSLS; SYASS; YASSY	NSYMP; SKFKK; SPRSP	AASLT; EGRLP
HIV-1	NSSSG; QYLAL	GGNSS; QYLAL; SSSPA	GSSRS; QYLAL	QYLAL	AASLT
HPV-B19	PPVPP; VNSVS	AGGGG; GGGGS; SSFQL; SSSSS	SSFQL	–	–
IVA (H1N1)	GGEVR	HSTQI	–	–	–
IVA (H5N1)	GGEVR	HSTQI	–	–	–
IVA (H7N7)	GGEVR; GSPPV	HSTQI	–	–	TPLGS
FLUBV	–	–	–	–	–
FLUCV	PMSHG; ASLGL	ASLGL; LQATA	–	–	RLPKG
MeV	–	AALQR; AGSSG; LAALQ	ASLGL	–	–
RV-A	ALNRR; LEDPG; LHSAG	AASST; IAASS; SYYTN	AALQR; AGSYP; AHPPT; HPPTS; LAALQ; PSSSD; SAGSY	–	–
RV-C	YIPSY	–	AASSI; KIMKN	–	–
RV ADRV-N	ISSVQ	–	PQSPA; SAASS	ASLGL	–
RUBV	AELAA; GRALS; RAELA	AELAA; RAELA; SASPP	–	–	–
VACV	FKKLM; ISSVQ; SYIPS; SSPYI	IVNGK; LEPEI; LPVST; NGKPK; PTQTP; PVSTA; SSSSS	AELAA; RAELA; TEPEV	–	ASLTP; TNAAS; YYPEL
HHV-3	DSEKS; GNAGS; LGYPY	ASPPC; GTSSS; IAASS; PSSAA; PVSAP; QPPSG; SGSSP; SPPCA; SSSSS; VSAGT	GSLQH; KALNP; LENSA; NGKPK; PGSLQ; QPEKE; RSKIK; SSINS; SSSDP; SSYLE; YCSPT; YSYAS	KTTVI; VIENG; HESDP	SLTPL
ZIKV	–	IRIVN	HPPTS; TSAAS; VFDRR; YTSAA	DDTDQ; IENGE; MPPNS	RLPVP

a *Number and percentage of pentapeptides shared with the 25 viral proteomes are reported in parentheses*.

At a first glance, the following points become apparent when considering Table 1:

- the neural proteins, including the neuronal regeneration-related protein NREP, have pentapeptides in common with all viral proteomes, excluding Borna disease virus and Influenza B virus;
- DLX2 is the main target of the peptide sharing by being 49% the level of DLX2 pentapeptide similarity to the 25 proteomes, i.e., 159 out of 324 DLX2 pentapeptides are shared with the viral proteomic ensemble;
- the peptide sharing mostly occurs with herpesviruses in general, and with HCMV in particular. Instead, the peptide sharing with HeV, HBV-C, RV-C, and RV ADRV-N is restricted to a few pentapeptides thus indicating a scarce contribution of such infectious agents in crossreactivity-triggered DLX alterations and consequent neurological manifestations;
- the viral pentapeptide distribution is not stochastic. For example, Vaccinia virus pentapeptides represent 27% of NREP peptide sharing (i.e., 3 out of 13 pentapeptides) and 4.4% of the DLX2 peptide sharing (i.e., 7 out of 159 pentapeptides);
- the extent of the peptide sharing is independent of the virus protein length. For example, Rubella virus (3,179 aa) and the eleven times longer HHV3 (35,782 aa) share exactly the same number of pentapeptides—namely, three—with DLX1 protein;
- quantitatively, the extent of the peptide sharing is unexpectedly high compared to the mathematical expected value of the pentapeptide sharing between the five neural proteins and the 25 viral proteomes. The expected value can be calculated as follows: given two protein entities (for example, DLX1, and the set of 25 viral proteomes) and assuming that all aa occur with the same frequency, the expected probability for the two entities to share a pentapeptide is expressed by the formula
  $$mn/N^{2}$$
  where: *m* is the number of pentapeptides present in the DLX1 protein (i.e., 251); *n* is the number of pentapeptides present in the set formed by the 25 viral proteomes (i.e., 418,854), and *N* is the number of all possible pentapeptides. Since each residue can be any of 20 aa, then N is 20^5^ (i.e., 3,200,000). Developing the equation, the expected pentapeptide sharing between DLX1 and the 25 viral proteomes is equal to 1,02668314453125e-5 or, practically, zero.

### The immunologic potential of the viral vs. DLX proteins peptide overlap

The pentapeptide matching between viral and neural proteins illustrated in Table 1 has an experimentally documented immunologic potential. As a matter of fact, exploration of the Immune Epitope DataBase (IEDB; www.immuneepitope.org; Vita et al., 2015) shows that almost all shared pentapeptides are also part of hundreds of epitopes that have been experimentally validated as immunopositive in humans. Using the epitope aa length as a filter, Table 2 is restricted to n-mer sequences with *n* < 12.

**Table 2:** Epitopes experimentally validated as immunopositive in the human host and containing pentapeptides common to the analyzed neural and viral proteins.

**IEDB ID^a^**	**Epitope sequence^b^**	**IEDB ID^a^**	**Epitope sequence^b^**	**IEDB ID^a^**	**Epitope sequence^b^**
38471	lpfEKSTVm	452745	appPALPPk	504154	aAELAAaaal
52131	vvpiASLTPy	453106	dLQATAldlew	504458	anGGGGGg
55320	rprGEVRFl	453171	eaLAALQaan	505711	grAGGGGPg
59401	sLPPPGtrv	454071	GGGGSrselvi	507555	PPVPPGtpmip
59519	SLTPLlfnydva	455984	LASGTpsani	507767	rkKAYADfy
78372	ELAASaivg	456139	lgtASLAQv	508461	sshsNSSSSsl
103335	lALEGSlqk	456387	llspGGPGStl	509354	daAASLTv
120627	iLPPPGy	456448	lnkSRPLGae	509896	LPPPGlti
124186	gftpGGGGS	458627	ryLAALQll	512096	ASSYLsltpeqw
131762	yaASSYL	459338	spdlgHSTQI	515359	esvaAALSPlg
146495	phvpPPVPP	459581	spSFSRPast	517427	GRLPVPkevnr
157857	rlGGAALprv	459696	sspEDPGAev	520364	krleKLLKQal
159292	plALEGSlqk	463283	aPALPPpaf	533287	phlgPPVPP
162373	gqrkSASSF	465679	glffPGSGGvit	542463	gqkPSSSDtf
162661	kiKEVTEev	466693	ipAGSSGSkflal	543080	iwlpFPVLL
179892	rpvPVSTAr	467021	iYIPSYfdf	544315	NSSSSstdsetlry
179919	vrpvPVSTA	467100	iyqGSSGSyf	544506	PVPPGgsrsnf
202757	asttRAELAy	468008	lPALPPslv	545645	tfSVSSHlf
218741	rvtYPAKAk	468164	lpPPPGSpl	551934	GGGGGggrfs
219023	sehGGGAGnnw	468966	nfGGGGGnf	552106	glGSGGSir
219569	stAHPPTgk	469915	rfiapAASLGf	555776	rsGGGGGggl
219711	SVSSHlfthk	470483	slAGSSGpga	561784	tPAGGGfpr
222414	gedaGGGAGrel	470910	spiiGSPPV	563865	htfNSSSSqy
222458	gEGSALeksl	471048	SPPVPgpsaal	570436	apgpaPPVSA
226892	rrlgPVPPGl	471225	SPVSGgvnl	570600	aPSSAAwvqtl
236284	qrkKAYADf	473075	vyAALQRqll	574225	kPALPPasl
239392	allAGGGGPpak	473232	vymLAASLl	574257	kplsSLTPLia
239680	fSASLAphfnsl	473386	vYYPELfvw	574258	kplsSLTPLiaa
241439	rRLPVPrak	474572	AALQRqll	574430	KRSKIksfvk
423867	afSLTPLey	474610	AASSIqrvl	576991	rqavGGAGPp
424337	ffdPAGGGd	475051	aeilGSGAGi	577112	RRFQKnihr
424618	fNSSSSfry	475122	AELAAhepai	581699	ahalnDSLVAl
425055	gsnfGGGGSy	475184	aeNAGSYsl	584847	kkkagPGSLQk
425181	gyGGGGPgy	478174	fpQPEKEsf	587586	PTSASrksl
425535	ifsgAASLGy	478359	geaGGGAGl	587901	rahPALPPl
426679	PAGGGdpily	478463	gelPAGGGl	588460	riAKAYAan
426734	qfGSALAhfy	478519	getGGGGGSal	585095	kplsSLTPL
427287	sifsgAASLGy	478693	gLNSPVligk	590425	tRLPKGavlyk
427412	snfGGGGSy	480790	KLLKQvdfl	589031	RRFQKtknll
427447	sSASLAasy	483709	qypGSALAl	590702	vhGRLPVhgv
427741	tffdPAGGGd	483949	reqVRFNGf	597556	KAYADfyrny
427753	tflnPALPPgysy	484016	rgilkrNSSSSstds	599267	lSSELRsvlvm
428949	YYPELafqf	484222	rlGGGGSpr	605152	hrLAALQhrl
435141	GRLPVahpgt	484341	RLPKGavlyk	605536	qrkKAYADfy
436575	ASLGLkfnk	484374	rlrlGGAAL	605589	rqiAKAYAr
437241	feqLAALQi	484531	rprGGGAGgssv	606593	aPALPPaaall
437831	gtPAGGGfpr	485822	sEVTEPdhpvl	608552	GRLPVvvplh
442711	aptkPQSPA	485924	shhPPVSAf	611431	RRFQKtknl
443226	dPPPGShvi	486263	sPAGGGaeal	614993	eaGSSRSqei
444635	GTSSSaasslk	486272	spaNSSSSl	615286	ehlGTSSSl
444517	GRLPVglsl	486452	sptSSASSF	616774	ghfpQPEKEsf
445920	kRLPVPesitgf	486557	SSASSsvtdly	617119	grwsGGSGA
446133	lefEGGEVsl	486749	stiNSSSSvvhk	617562	HHHHHpvspa
446393	lpRLPVPav	487343	teqdPGSASa	619990	krleKLLKQ
464008	dASLTPwtv	488260	vlrGGGGSpr	619991	krleKLLKQa
446422	lpswgRAELAl	488347	vpsGSSGSl	628491	YPAKAkgtf
447091	qHLQATgvsl	488735	yaASSYLsl	629394	alYYPELyilk
448056	rvPPVPPnv	491894	grSLTPLsl	632427	iGSLQHiksr
449644	vlfgKALNPk	492854	kRLPVPesi	633424	kSLTPLqw
451656	aeADSEKarll	495589	tRLPKGavly	645286	ispPPVSAv
452446	alSASLArv	496005	wrlrlGGAAL	647258	lqnLENSAf
452673	aPGSASgpl	496112	yrgvLNSPV	647281	lqRLPVPal

a *Epitopes listed according to the IEDB ID number. References at www.immuneepitope.org/*.

b *Peptide sequences common to neural proteins and potential viral pathogens in capital*.

### Comparative DLX transcript expression in fetal and adult human brain

**Figure 1:**
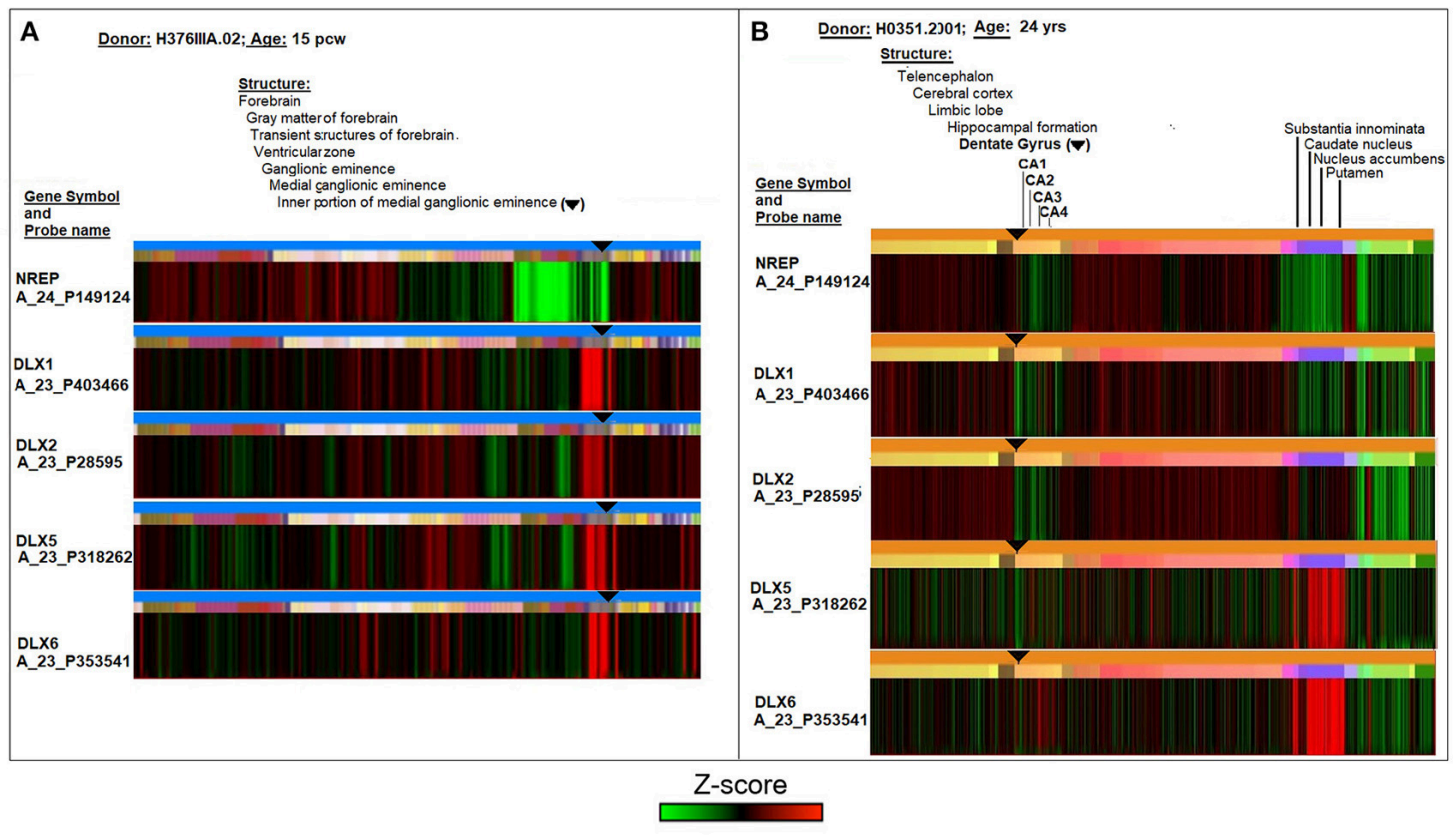
Comparative analyses of DLX1, DLX2, DLX5, and DLX6 transcript expression in fetal (15 post-conception weeks, pcw; **A)** and adult (24 years; **B**) human brain. The symbol ▾ localizes neurogenic areas of developing and adult brain. The figure assembles images and data from the Allen Institute. Further details on donors, DNA probes, complete transcriptome profiles, and methodology can be found at http://www.brainspan.org/ and http://human.brain-map.org/ (Lein et al., 2007; Miller et al., 2014).

A comparative analysis of DLX expression in fetal and adult neurogenic areas of the human brain was conducted using the online database and resources of the Allen Institute for Brain Science (Lein et al., 2007; Miller et al., 2014). Figure 1 reports laser microdissection microarray analyses showing that the transcript expression of the four TFs ranges from medium to high in the fetal transient structures of forebrain (ventricular zone and ganglionic eminence) (Figure 1A), and reaches the lowest but still detectable value in the adult neurogenic dentate gyrus (Figure 1B, subareas CA1, CA2, CA3, and CA4). Notably, only DLX5 and DLX6 appear to be expressed in basal ganglia of adult brain, that is, substantia innominata, caudate nucleus, nucleus accumbens, and putamen (Figure 1B). The control neuronal regeneration-related protein NREP is widely expressed through almost all fetal and adult brain areas, except the fetal ventricular zone and ganglionic eminence (Figure 1A) as well as the dentate gyrus area in the adult brain (Figure 1B).

### DLX protein expression in fetal and adult human brain

On the whole, Figure 1 theoretically supports the possibility that the cross-reactivity scenario outlined in Tables 1, 2 may occur in neurogenic areas in the fetal life of an individual and then possibly recur in adulthood. However, from an immunological point of view, a condition that is necessary for the cross-reactivity hypothesis to be biologically plausible depends on sufficient DLX antigen expression in the brain. In other words, data of Figure 1 need to be substantiated in a protein context.

Actually, few data are available on DLX protein expression in humans. Rakic and Zecevic (2003) studied DLX expression in the late human embryonic period (Carnegie stages 19–20) and showed that DLX2 protein was widely distributed through the ganglionic eminence and dorsal telencephalon. Moreover, immunocytochemistry based on a pan-DLX antibody that recognizes DLX 1, 2, 5, and 6 revealed that, in the developing brain, 11 gestational week, DLX protein expression is present in all cortical layers, including layer I and the subpial granular layer (SGL). Almost all small GABAergic cells of the SGL were labeled with the pan-DLX antibody. Successively, using the same pan-DLX antibody, Jakovcevski et al. (2011) showed labeling of the neocortical VZ/SVZ and of the cortical plate in human fetal forebrains during the first half of gestation.

**Figure 2:**
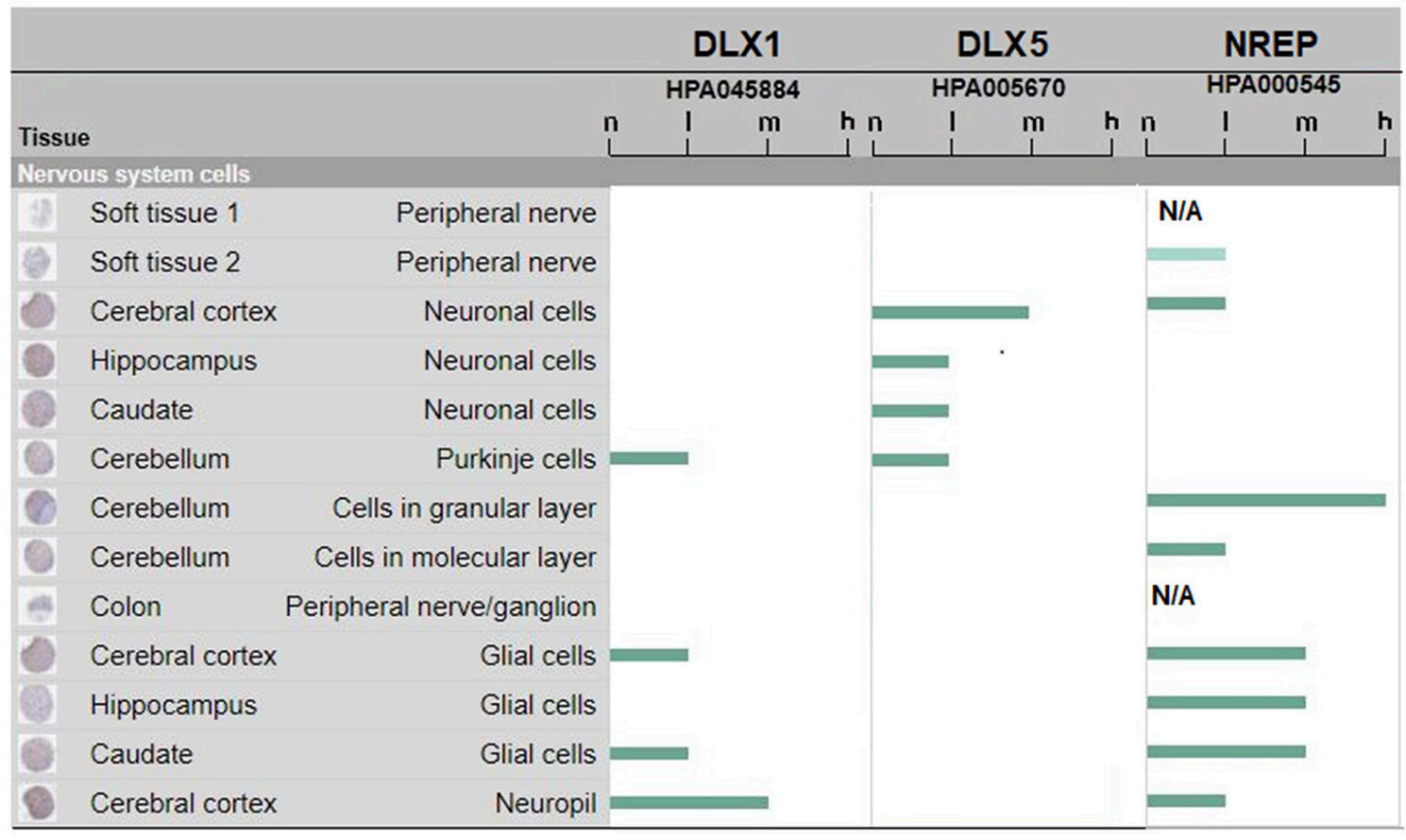
DLX1, DLX5, and NREP protein expression in adult human brain. Estimate of the protein expression are: not detected (n), low (l), medium (m), or high (h). Data for DLX2 and DLX6 proteins were not available or pending. The figure assembles images and data from www.proteinatlas.org (Uhlén et al., 2015; Thul et al., 2017).

Such experimental results obtained in human fetal developing brain are flanked by data collected from the Human Protein Atlas (https://www.proteinatlas.org/on DLX1 and DLX5 protein expression in the adult human brain. The data are shown in Figure 2. It can be seen that human DLX1 and DLX5 have a protein expression from low to medium level, that is, sufficient to sustain immune cross-reactions. No protein expression data were available for DLX2 and DLX6 proteins. The control NREP had the highest levels of protein expression (from medium to high in the cerebral cortex and the cerebellum).

In essence, we found a vast and unexpected peptide sharing between DLX proteins and numerous infectious agents that constellate human life, from prenatal time to adulthood. The peptide platform defined in Table 1 has also a high immunologic potential, as documented in Table 2, so that, on the whole, data from Tables 1, 2 show the existence of a wide immunologic peptide platform common to viral and human DLX proteins. Moreover, data on protein expression from literature (Rakic and Zecevic, 2003; Jakovcevski et al., 2011) and Figure 2 (www.proteinatlas.org; Uhlén et al., 2015; Thul et al., 2017), although few in numbers and fragmentary, support the possibility that mild, subclinical anti-DLX autoimmune damage in the fetal brain structures evoked by maternal viral infections (and consequent maternal immune activation) may be followed in the adult brain by additional damage after a second encounter with the same pathogen or novel infection with a different agent sharing the same epitopic sequences.

In this context, different immunological pathogenic mechanisms might be theoretically account for the neuronal damage according to the type of immune response, i.e., humoral vs. cell-mediated, and the timing of infection-induce maternal immune activation in relationship to the expression patterns of DLX proteins in the fetus (see Figure 1). The main hypothesis that we considered here relates to maternal infection and consequent immune activation that may also be antecedent to pregnancy and are followed by antibody–mediated neuronal damage in the fetus due to cross-reactions with DLX proteins. In such a scenario, the passage in the fetus of maternal memory B cells (Vernochet et al., 2005, 2007) against epitopes shared between the pathogen(s) and DLX proteins might induce an immune response targeting the developing nervous systems, where DLX proteins are expressed early (see Figure 1). Cellular damage from (auto)antibodies targeting intracellular antigens, like the DLX family of TFs may be, not only plays a pathogenic role in a variety of autoimmune diseases (Racanelli et al., 2011) but even represents a promising therapeutic strategy for cancer treatment (Weisbart et al., 2012; Wang et al., 2015; Chan et al., 2016). The notion that autoantibodies can penetrate living cells is not new. Alarcon-Segovia et al. (1978) showed that antibodies can penetrate living cells. In more recent years, more evidence has accumulated showing autoantibody penetration into different types cell, including neurons, and proposing mechanisms that may explain a pathogenic role of internalized immunoglobulins in autoimmune diseases (Koren et al., 1995; Koscec et al., 1997; Portales-Pérez et al., 1998; Ruíz-Argüelles et al., 1998, 2003, 2007; Ronda et al., 2002; Proulx et al., 2009). Moreover, a nuclear-penetrating lupus anti-DNA autoantibody, 3E10, has been found to inhibit DNA repair and selectively kill certain cancer cells that are highly vulnerable to DNA damage (Weisbart et al., 2012), and, of special importance, nuclear-penetrating anti-dsDNA autoantibodies could possibly function as a pathogenic autoimmune factor for lupus nephritis (Im et al., 2015). Bearing even more relevance to the present discussion, antibodies targeting intracellular antigens, like for instance the glutamic acid decarboxylase, appear to be also involved specifically in neuropsychiatric disorders, like CNS lupus (Karimifar et al., 2013), limbic encephalitis (Matà et al., 2008), schizophrenia (Najjar et al., 2012), and autism (Rout et al., 2012). Indeed, the glutamic acid decarboxylase isoforms (Gad1 and Gad2), which regulate GABA synthesis from the excitatory neurotransmitter glutamate and whose expression is activated by DLX1 and/or DLX2 (Stühmer et al., 2002a,b; Le et al., 2017), share numerous pentapeptides with the 25 viral proteomes (see Supplementary Table 1). Hence, a scenario emerges where immune responses following infections might cause a cascade of multiple crossreactions at multiple levels (i.e., DLX, GAD) of the intracellular mechanisms regulating the function of GABAergic neurons and altering the excitation and inhibition ratio, which is necessary for normal neural circuit function and whose imbalance contributes to neurodevelopmental diseases (Kang, 2017; Maffei et al., 2017; Ye and Kaszuba, 2017; Catavero et al., 2018; Garret et al., 2018).

On the other hand, a cell-mediated mechanism could also theoretically be implied in the cross-reactive immune-mediated subclinical damage of the fetal nervous systems, since memory T-cell trafficking between mother and fetus is also a well-known phenomenon (Jeanty et al., 2014). Nevertheless, the hypothesis of a cell-mediated response would need to take into account the late MHC expression in the fetal CNS (Elmer and McAllister, 2012; Zhang et al., 2013, 2015; McAllister, 2014) that might not sit well with the very early pattern of expression of the DLX-proteins in the fetus seen in Figure 1. However a later cell-mediated damage, and even the possible occurrence of both humoral and cell-mediated responses at different stages of the fetal neural development, can still be hypothesized.

Based on data from Figure 2 and, consequently, confining our discussion to DLX1 and DLX5 proteins, we observe that infection-induced immune cross-reactions might have functional, spatial, and temporal implications:

- Functionally, infection-induced immune cross-reactions would affect two TFs that, according to numerous studies, are implicated in crucial functions and fundamental processes during neurodevelopment and adult neurogenesis, and are potentially relevant to language competence and other higher cognitive functions (see Box 1);
- Spatially, infection-induced immune cross-reactions would damage brain structures where adult neurogenesis occurs and that are involved in the neural circuitry of language and memory, and in cognitive and emotional functions (Ming and Song, 2011). Altered human neurogenesis is linked to neuropsychiatric conditions and to impaired cognition (Aimone et al., 2014). Also, alterations of the SVZ and hippocampus have been specifically related to some of the pathogenic and symptomatic aspects of schizophrenia (Reif et al., 2006, 2007; Duan et al., 2007; Toro and Deakin, 2007; Christian et al., 2010; Aimone et al., 2014; Allen et al., 2016; Kang et al., 2016; Yun et al., 2016; Iannitelli et al., 2017; Terrillion et al., 2017).
- Temporally, infection-induced immune cross-reactions suggest a two-hit model that, depending on the DLX protein expression profiles, comprehends targets allocated in two time-windows in the life of an individual with a subclinical damage in fetal life and clinical onset in adulthood.

Box 1 **DLX1 and DLX5 functions and relevance to neuropsychiatric disturbances.**

**DLX1:**

- regulates the development of the ventral forebrain, craniofacial patterning, and the early diencephalic subdivision (Eisenstat et al., 1999; Merlo et al., 2000; Letinic and Rakic, 2001; Letinic et al., 2002).
- regulates neurite maturation and migration (Cobos et al., 2007) and interneuron differentiation (Wonders and Anderson, 2005).
- regulates the fate switch between cortical and striatal interneurons: cells that ordinarily would become cortical interneurons transform toward a subtype of GABAergic striatal interneurons, thus reducing glutamatergic input to the hippocampus (McKinsey et al., 2013).
- its loss leads to subtype-specific loss of inhibitory interneurons with a reduction in inhibitory currents and generalized seizures in mice (Cobos et al., 2005; Jones et al., 2011).
- contributes to promote cortical interneuron migration from the basal forebrain by direct repression of the semaphorin receptor neuropilin-2 (Le et al., 2017).
- when altered, might be related to Mowat-Wilson syndrome (McKinsey et al., 2013).
- is downregulated or altered in autism spectrum disorders (ASD; Liu et al., 2009; Voineagu et al., 2011; Benítez-Burraco et al., 2016).
- has low thalamic expression in psychosis (Kromkamp et al., 2003).
- has been proposed as a language-associated protein (Boeckx and Benítez-Burraco, 2014; Benítez-Burraco et al., 2016; Murphy and Benítez-Burraco, 2016, 2017) and relates to language deficits in schizophrenia (Murphy and Benítez-Burraco, 2016).

**DLX5:**

- promotes neuronal differentiation in SVZ (Shu et al., 2010).
- its loss leads to defective neuronogenesis (Perera et al., 2004).
- contributes to convert fibroblasts into induced GABAergic interneurons (Colasante et al., 2015).
- regulates development of peripheral and central components of the olfactory system (Long et al., 2003).
- is a candidate genes for autism (Nakashima et al., 2010).
- is involved in Rett syndrome (Itaba-Matsumoto et al., 2007; Itoh et al., 2007).
- participates to the regulation of the expression of the glutamic acid decarboxylases (Stühmer et al., 2002a).
- its loss preferentially reduces the number of mature parvalbumin- interneurons (Wang et al., 2010).

## Conclusion

In synthesis, the present study confirms previous reports (Kanduc et al., 2008; Lucchese et al., 2014; Lucchese, 2017) and supports the hypothesis that an autoimmune connection exists at the molecular level among infections, autoimmune reactions and neuropsychiatric disorders. Such a connection implies a vast viral vs. neural proteins peptide overlap and operates through cross-reactivity mechanisms. To test this hypothesis *in vivo*, the possibility of obtaining animal models of neuropsychiatric disorders by immunizing pregnant animals with DLX proteins could be examined. Moreover, analyses of sera from human patients with neuropsychiatric diseases, for example schizophrenia, are warranted to measure immunoreactivity against the peptides shared between viruses and DLX proteins. Possibly, the results of these joint basic and clinical *in vivo* approaches might also help design new therapeutic approaches in neuropsychiatry.

## Author contributions

All authors listed have made a substantial, direct and intellectual contribution to the work, and approved it for publication.

### Conflict of interest statement

The authors declare that the research was conducted in the absence of any commercial or financial relationships that could be construed as a potential conflict of interest.
