# Supplementary material for: Peptide Sharing Between Viruses and DLX Proteins: A Potential Cross-Reactivity Pathway to Neuropsychiatric Disorders
Source: Front Neurosci. 2018 Mar 21;12:150. doi: 10.3389/fnins.2018.00150 (PMC5871705; doi:10.3389/fnins.2018.00150)
Supplement: Supplementary file 1 [file Table1.DOCX]

# Supplemental Table 1. Pentapeptide sharing between twenty five viral proteomes and glutamate decarboxylases 1 (GAD-67) and 2 (GAD-65)

| **Virus** | **Viral Pentapeptides shared with** | |
| --- | --- | --- |
|  | **GAD-67 (119; 20%)^1,^** | **GAD-65 (145; 25%)^1,^** |
| BDV | ⎯ | ⎯ |
| EBV | GFLQR; FLQRT; AFKER; TETDF; PESLE; TVYGA; LELAE; KIKNR; LRGVP; GVPDS | SPGSG; GSEDG; ARAWC; RAAAR; VGLAA; PGGSG; ALPRL; SHFSL; TVYGA; DKALQ; LELAE; YIPPS; YQPLG |
| HeV | RTETD; GLDII; ILEAK; YDTGD | NLEEI; LEEIL; ILEAK; DPLLA; PLLAV; YDTGD |
| HBV-C | PSSSA; GWSSK; IPQSL | ⎯ |
| HCVH | ⎯ | ⎯ |
| HCMV | SSSAT; SSATS; TSSNA; SSNAG; RLVSA; LFARD; ARDLL; ARDLL; LLPAK; LEVVD; TLKYG; VRTGH; LTSTA; YSIMA; AARYK; KLVLF; GAALG; AGTTV; AGTTV; GGGLL; SRKHR; RKHRH; LRGVP | SPGSG; LCALL; AARKA; TDLLP; DGERP; VKSFD; TTLKY; LTSTA; AALPR; AALGI; PSDLE; SDLER; ERRIL; RILEA; AGTTV; LAVAD; GGGLL; VDKCL; SLRTL; RTLED; DNEER |
| HHV1 | ARFRR; EQTVQ; VDILL; PGGAI; GAALG; GAALG; ALGFG; VPFYV; LYAKI; RGVPD; GVPDS | PRAAA; RAAAR; RPTLA; TLKYA; GLAAD; SGDGI; PGGAI; GAAAL; AAALG; SATAG; LLAVA; PPSLR |
| HHV2 | EQTVQ; VDILL; PGGAI; LEAKQ; HVDAA; ERANS; RGVPD | AEKPA; PPRAA; PRAAA; RAAAR; YVVKS; TKVID; NELLQ; TLKYA; PGGAI; AALPR; LERRI; LEAKQ; VSATA; SATAG; HVDAA; ERANS; PPSLR |
| HHV6A | SKNLL; KNLLS; PAKNG; LKYGV; KYGVR; LKKMR; KLVLF; ERGKI; PKIKA | GGIGN; AARKA; PTLAF; YAIKT; VFVLL; LKKMR; GMAAL; MAALP; HFSLK; DSVIL; SDLER; DLERR; GVPLQ; DKALQ |
| HHV6B | SSTPS; SKNLL; KNLLS; VVDIL; NYVRK; SLEQI; LKYGV; LKKMR; ERGKI; SPQRR | SPGSG; AARKA; LHATD; PTLAF; ELLQE; YAIKT; VFVLL; LKKMR; FSLKK; DSVIL; SDLER; DLERR; GVPLQ; DKALQ; NEERM |
| HIV-1 | LELAE; KIKAL | LELAE |
| HPV-B19 | LSDHP; ANSVT | QNLEE; KGAAA; ANSVT |
| IVA (H1N1) | PSSSA; SLRGV | ⎯ |
| IVA (H5N1) | PSSSA | LERRI |
| IVA (H7N7) | PSSSA; VGWSS | ⎯ |
| FLUBV | LGLKI; LPAKN | AALGI |
| FLUCV | LELSD | ⎯ |
| MeV | KLGLK; LLEVV; AGAAL; GAALG; ESGTT | ⎯ |
| RV-A | ILLNY; ANSVT | TGGIG; GGIGN; ANSVT; LYNII; EYGTT |
| RV-C | LELAE; IEEIE | TGGIG; LELAE; IEEIE |
| RV ADRV-N | REEFE | TLAFL; NILLQ |
| RUBV | ⎯ | GTARA |
| VACV | GADPN; DILLN; ILLNY; HPHQL; FNQLS; TGLDI; LIKCN; SRKHR; PKIKA; IKALM; VISNP; IEEIE | SGDSE; TLKYA; LKYAI; FNQLS; LLEYV; EYVTL; MREII; RLIAF; DERGK; SDLER; LVSAT; KHYDL; YDLSY; VDVFK; DVFKL; NIIKN; VFDGK; RTLED; VISNP; IEEIE |
| HHV-3 | STPSS; LSTGL; TYEIA; SIMAA; SRKHR; RGVPD; SDIDF | CALLY; RPTLA; YAIKT; LSTGL; LAADW; TYEIA; YVTLK; MREII; KGTTG |
| ZIKV | GAALG | MREII |

^1^Number and percentage of pentapeptides shared with the 25 viral proteomes are reported in parentheses
